# Caffeine and attentional control: improved and impaired performance in healthy older adults and Parkinson’s disease according to task demands

**DOI:** 10.1007/s00213-021-06054-9

**Published:** 2022-01-10

**Authors:** Kanch Sharma, Sean James Fallon, Thomas Davis, Scott Ankrett, Greg Munro, Gary Christopher, Elizabeth Coulthard

**Affiliations:** 1 ReMemBr Group, Level 1, Learning & Research, Southmead Hospital, Bristol, BS10 5NB UK; 2 College of Health Sciences, Amoud University, Borama, Somaliland; 3 National Institute for Health Research Bristol Biomedical Research Centre, University Hospitals Bristol NHS Foundation Trust and University of Bristol, Bristol, UK; 4 Faculty of Health and Applied Sciences, University of the West of England, Coldharbour Lane, Bristol, BS16 1QY UK

**Keywords:** Caffeine, Attention, Yerkes-Dodson, Withdrawal, Parkinson’s disease

## Abstract

**Introduction:**

Caffeine is frequently consumed to boost goal-directed attention. These procognitive effects may occur due to the adenosine-mediated enhancement of monoamines, such as dopamine, after caffeine administration. As such, caffeine’s beneficial effects may be altered in conditions such as Parkinson’s disease (PD). However, whether caffeine improves cognition, and at what cost, has not been experimentally established in patients with neurodegenerative disease.

**Methods:**

Single-dose trials to probe cognitive effects of caffeine are often confounded by short-term caffeine abstinence which conflates caffeine’s effects with treatment of withdrawal. Using a placebo controlled, blinded, randomised trial design, we assessed the effect of 100 mg of caffeine across well-established tasks (Choice reaction time, Stroop Task and Rapid Serial Visual Presentation Task; RSVP) that probe different aspects of attention in PD patients (*n* = 24) and controls (*n* = 44). Critically, participants withdrew from caffeine for a week prior to testing to eliminate the possibility that withdrawal reversal explained any cognitive benefit.

**Results:**

Caffeine administration was found to reduce the overall number of errors in patients and controls on the Stroop (*p* = .018, *η*^2^_*p*_ = .086) and Choice reaction time (*p* < . 0001, *η*^2^_*p*_ = .588) tasks, but there was no specific effect of caffeine on ignoring irrelevant information in the Stroop task. On the RSVP task, caffeine improved dual item accuracy (*p* = .037) but impaired single item accuracy (*p* = .044). Across all tasks, there was little evidence that caffeine has different effects in PD participants and controls.

**Conclusion:**

When removing withdrawal effects as a factor, we demonstrate caffeine has beneficial effects on selective attention but is a double-edge sword for visual temporal attention and would need careful targeting to be clinically useful.

**Supplementary Information:**

The online version contains supplementary material available at 10.1007/s00213-021-06054-9.

## Introduction

Impairments in goal-directed attention are a frequent problem in older adults (Zanto and Gazzaley 2014). Normal age-related cognitive decline can be a significant source of distress and economic burden, but these problems become magnified in neurodegenerative conditions like Parkinson’s disease (PD). Although symptomatic treatment of PD focuses on motor deficits, cognitive deficits can be disabling, even in the early stages with mild cognitive impairment affecting 19 to 55% (Goldman and Litvan 2011). The increased level of cognitive deficits in PD patients is partly explained through the increase in dopaminergic degeneration this group displays compared to normal ageing (Kaasinen and Rinne 2002). However, there is also pathology to noradrenergic, serotonergic and cholinergic systems (Bohnen & Albin 2011; Kish et al. 2008; Vazey & Aston-Jones 2012), again to a greater extent than seen in normal ageing, which may also be responsible for driving cognitive deficits (Kehagia et al. 2012). In this study, we assess the ability of caffeine to act as a novel enhancer of goal-directed attention in PD patients and healthy older adults. Specifically, we address the hypothesis that caffeine improves all forms of goal-directed attention or only a subset of attentional functions, and whether the pattern of attentional gains is similar in patients and controls.

Caffeine is often overlooked as a cognitive enhancer for healthy older adults and PD due to its widespread habitual use across society. However, caffeine is safe and easily traverses the blood–brain barrier to exhibit its main neurochemical effect: blocking the effects of the endogenous neuromodulator adenosine which eventually triggers release of excitatory neurotransmitters such as glutamate, norepinephrine, acetylcholine and dopamine (Koppelstaetter et al. 2008; Ribeiro & Sebastião 2010). Neuroimaging has also revealed that caffeine enhances bilateral activity in the striatum and functional connectivity in fronto-striatal regions in older adults (Haller et al. 2013). Therefore, there are several routes through which caffeine could modulate cortical arousal and benefit cognition, potentially counteracting the effect of neuronal depletion found in PD and ageing.

The effects may also be different in PD. Pharmacologically, compared to age-matched health controls, PD is associated with a decrease in adenosine A2A receptors in the dorsal striatum, an increase in the substantia nigra pars reticulata, but with no change in any other brain regions (Hurley et al. 2000). Adenosine A2A receptors are also co-localised with dopaminergic D2 receptors on GABAergic neurons and have antagonising effects (Benarroch 2008; Ferré 2008; Fredholm & Svenningsson 2003). Striatal D2 receptor activation forms part of the striatopallidal indirect pathway which is concerned with suppressing motor activity, in balance with the direct pathway in enhancing voluntary motor actions (Svenningsson et al. 1999). Adenosine A2A receptor activation theoretically suppresses GABAergic neuronal inhibition of the indirect pathway and should therefore improve movement in PD by restoring some balance between the direct and indirect dopamine pathways (Mori & Shindou 2003), although more research is needed. Thus, A2A receptor antagonists such as caffeine should exert a similar effect to dopamine agonists and could function as an add on to conventional levodopa therapy in PD (Vuorimaa et al. 2017). However, whether these effects extend to the cognitive domain has not been fully explored.

Caffeine may not affect attention in a monolithic fashion. Attention is not a unitary construct but has been argued to contain three independent systems. Functionally, attention can be distilled into a three-stage process: disengaging attention from its current target, shifting attention to a new focus and processing the new target (Petersen & Posner 2012). Voluntary orienting to visual information has been associated with control networks in dorsal frontal and dorsal parietal cortex which bias activity in the visual cortex to favour the processing of important over irrelevant stimuli (Corbetta et al., 2008; Grent- ‘t-Jong & Woldorff 2007).

Of these networks, the effect of caffeine on Stroop performance that maps on to the concept of executive attention has been the most widely studied. Several electrophysiological studies have found that caffeine can improve the neural markers of selective attention (Lorist et al. 1994, 1995; Ruijter et al. 2000) but the extent to which these translate into improvements in behaviour is unclear. For example, in the Stroop task, there are mixed findings concerning whether caffeine specifically improves performance in trials requiring selective attention (incongruent condition) compared to enabling non-selective performance enhancements to take place (Kenemans et al. 1999; van den Berg et al. 2020). PD patients have also been found to be impaired at the Stroop task (Brown and Marsden 1998), but whether caffeine ameliorates this deficit, and to the same extent as controls, remains to be explored. However, given the heterogenous nature of attention (Petersen and Posner 2012), declaring specific effects on attention function is difficult as performance on one task could be augmented through many different processes such as enhanced motivation or increased vigilance.

The effects of caffeine on Stroop performance, and its attentional pre-requisites, can be compared to tasks measuring simple and choice reactions times. These tasks assess the ability to sustain vigilance on a limited range of stimuli, representing alerting attention (Petersen & Posner 2012). In withdrawn consumers, acute caffeine has demonstrated beneficial effects on the speed of response and accuracy in both simple and choice reaction time (Smith et al. 2013); however, this effect was absent if sleep deprivation was an added factor (Rogers et al. 2005). Chronic caffeine use has not been demonstrated to improve choice response latencies (Judelson et al. 2005). Simple and choice reaction times are found to be impaired in PD due to cognitive, rather than motoric, difficulties (Kutukcu et al. 1999). The effect of caffeine on simple and choice reaction times seems largely unknown, but given caffeine’s adrenergic effects (Nehlig et al. 1992), a beneficial effect in this cohort (as with healthy controls) is likely.

Temporal constraints on attention have been extensively studied, particularly using the Rapid Visual Serial Presentation (RSVP) task (Raymond et al. 1992). The RSVP paradigm stresses the temporal capacity of visual selective attention to its limit, enabling characterisation of the efficiency at which information is analysed and encoded (Raymond et al. 1992). The participant is required to identify two targets when stimuli (letters) are presented in the same location in rapid succession. This task illustrates the phenomenon of the ‘attentional blink’ (AB), where identification of an initial target is followed by a refractory period preventing a second target being identified. RSVP performance has been compared between non-demented PD participants and aged-matched healthy controls; there was no difference in AB, suggesting the increased AB magnitude is secondary to age-related decline (Vardy et al. 2003). The duration of attentional blink has been found to be related to dopamine receptor levels in the striatum (Slagter et al. 2012) and administering dopamine to PD patients can decrease the size of the attentional blink in these patients (Slagter et al. 2016). Therefore, given that caffeine can modulate dopamine, via its effects on adenosine, RSVP performance should be affected by caffeine administration.

Brunye and colleagues investigated synoptic effects of caffeine on attention and found dose-dependent effects of caffeine on different aspect of attentional networks (Brunye et al. 2010). Following overnight withdrawal, intermediate doses of caffeine were associated with improved alerting and executive attention, but impaired orientating. In a similar vein to Brunye, a more recent study (Huertas et al. 2019) assessed the effect of caffeine versus placebo on all three attentional networks under the conditions of either rest or aerobic exercise. They showed that caffeine improved reaction times across tests of attention, but only in moderate users. Whilst these results point to differential effects of caffeine across attentional demands, there are several methodological flaws. Crucially, most studies which demonstrate a beneficial psychostimulant effect of caffeine have not fully withdrawn study participants from caffeine prior to testing (Warburton 1995). This has led to scepticism of caffeine producing a net benefit to users and the formation of the caffeine withdrawal reversal hypothesis (Bruce et al. 1991; James 1998; James & Rogers 2005; Yeomans et al. 2002), i.e. that caffeine consumed prior to full withdrawal, simply acts to ameliorate the fatiguing effects of withdrawal itself rather than produce an overall, net improvement in cognitive function.

The effect of caffeine on attentional networks in fully withdrawn participants has not been systematically tested. Here we seek to address this issue by examining the effect of caffeine on tasks that tax each of these networks. We selected neuropsychological paradigms that would individually probe each facet of the trinity of independent but interacting attentional networks, as described by the Posner-Petersen model (Petersen & Posner 2012). The Rapid Serial Visual Presentation (RSVP) paradigm evaluated the ability to align attention to a changing source of sensory input, reflecting orienting attention. The Stroop task was applied to evaluate top-down attentional control and the ability to focus attention selectively according to task demands, representing executive attention. Here we overcome the issue of withdrawal by using a prolonged caffeine withdrawal of 7 days. We also reduce the impact of practice effects on repeat testing on and off caffeine by performing baseline and 7-day testing on the experimental tasks before entering the placebo-controlled trial phase.

## Methods

Forty-two healthy elderly participants and twenty-four PD patients were recruited from a research volunteer database held in North Bristol NHS Trust. Participants all had capacity to give fully informed consent. Participants with any concomitant serious illness likely to interfere with cognitive or physical performance were excluded. Of 78 eligible healthy elderly participants screened, 44 were randomised and 42 were able to complete all 4 test sessions, whilst 2 participants could not adhere to caffeine abstention. Of 48 eligible PD patients screened, 26 were randomised and 24 were able to complete all 4 test sessions, whilst 2 patients could not adhere to caffeine abstention. No PD participants were on cholinesterase inhibitors or cognitive enhancers. All PD participants were on a dopaminergic therapy and none smoked (Table 1).

**Table 1:** Comparison of PD and healthy control participant demographics

	Healthy elderly	Parkinson’s disease
Participants	42	24
Age	73 (55–91)	67 (55–78)
Sex	18 male:26 female	15 male:9 female
Baseline MoCA	26.6 (23–30)	26.6 (20–30)
Habitual daily caffeine intake (mg)	104.1 (5–340)	112.3 (2.5–300)

### Testing protocol

A single-blind, cross-over trial compared 100-mg caffeine (Proplus) tablets dissolved in instant decaffeinated coffee, with instant decaffeinated coffee. The coffee was served with or without artificial sweetener as per patient preference but consistently given across the trial. Milk was not offered. The drink was served at a temperature range of between 50 and 60 °C, confirmed by measurement with a thermometer.

**Fig. 1:**
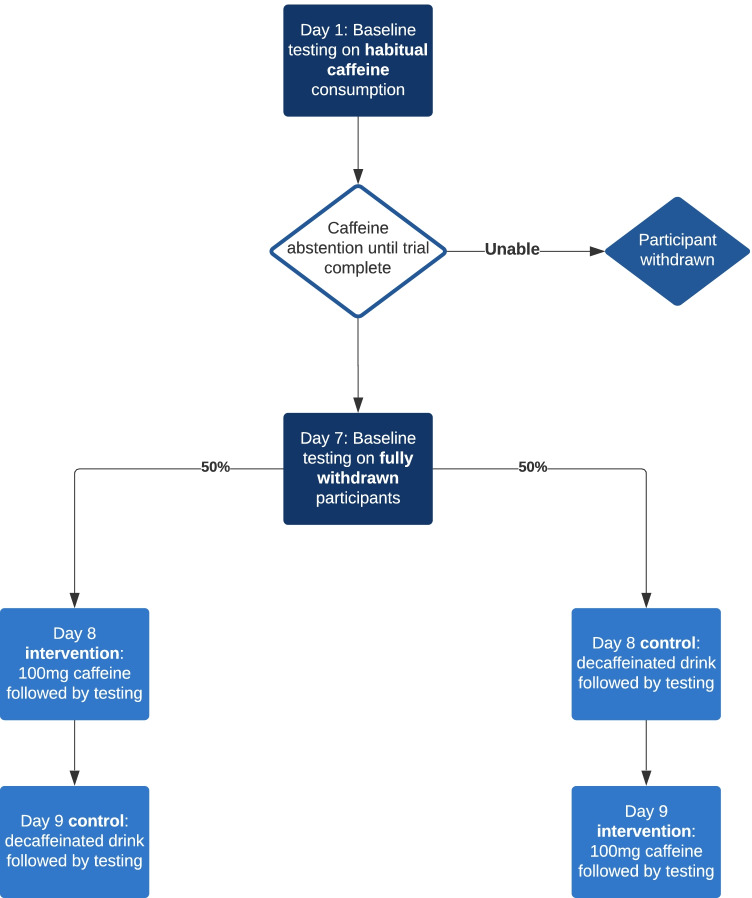
Testing protocol. From entering the trial, participants did not voluntarily consume caffeine except when part of the testing protocol

Participants attended for baseline testing on day 1 without any dietary caffeine restriction. Following testing they were given a supply of either decaffeinated coffee and/or decaffeinated tea to cover the trial duration (as per their consumption preference) and requested not to ingest caffeine-containing foods such as tea, coffee and chocolate for the remainder of the trial (9 days) but could freely consume the decaffeinated tea/coffee supplied to them. On day 7 (i.e. 1 week free from caffeine), participants repeated testing to assess for effects of caffeine withdrawal on attention and allow task familiarisation so that the effect of learning on subsequent performance was minimised. On day 8, participants received either caffeinated or decaffeinated coffee and testing started 60 min later. In the interim, participants would wait in a quiet waiting room with books and magazines for interest if desired. On day 9, the participants received the alternative type of coffee (caffeinated or decaffeinated whichever not already allocated) and began testing 60 min following consumption. Testing was performed within 15 min of the same time on all days. The task battery was performed in the same chronological order as presented below, for all participants on each visit (Fig. 1).

All neuropsychological paradigms were performed using pre-programmed tasks by Presentation software (Version 18.0 NeuroBehavioral Systems, Albany, CA, USA) which was run on a 15-inch Toshiba laptop running 32-bit Windows 7 pro or a 15-inch Dell laptop with 64-bit Windows 7 pro. A Cedrus RB-844 response box was used to record participant responses. The same laptop and response box were used across all testing sessions for an individual, to negate any intra-variability discrepancies as a result of computer hardware or software precision (Plant and Turner 2009).

#### Simple reaction time (SRT; Fig. Supplementary data 1A, *left*), i.e. a single response to a single stimulus

Each time a ‘red square’ (2 cm × 2 cm) was presented in the centre of a computer screen, the participant was required to press the corresponding ‘red’ coloured button on a free-standing response pad as quickly and accurately as possible. There was a variable fore-period prior to stimulus onset of between 1500 and 3500 ms and stimuli were displayed for 2000 ms. The task comprised 10 practice trials followed by 100 test trials and responses between 100 and 5000 ms were recorded. Responses outside this range were recorded as mistrials. The extracted metrics were mean reaction time, measured from the onset of the stimulus until the participant’s response on the response pad and mistrial rate. The extracted metrics were mean reaction time, measured from the onset of the stimulus until the participant’s response on the response pad and mistrial rate.

#### Choice reaction time

Choice reaction time (CRT; Fig. Supplementary data 1A, *right*) — there are two responses to two stimuli. Each time a ‘red square’ or ‘blue square’ was presented in the centre of the screen, the participant was required to press the corresponding ‘red’ or ‘blue’ coloured button on the free standing response pad as quickly and accurately as possible. There was a variable fore period prior to stimulus onset of between 1500 and 3500 ms and stimuli were displayed for 2000 ms. The task comprised 10 practice trials followed by 100 test trials and responses between 100 and 5000 ms were recorded. Responses outside this range were recorded as mistrials. If the incorrect colour was selected, the response was recorded as an error. Just like SRT, the dependent variable was mean reaction time, measured from the onset of the stimulus until the participant’s response on the response pad, mistrial rate and error rate.

#### Stroop test

Participants are presented with the name of a colour in a coloured font in the centre of the screen, and they must identify the colour of the font by pressing the corresponding button on the Cedrus RB-844 response box (Fig. S1B). There are two conditions. Congruent: In this condition, the colour name and the colour of the font are the same. For example, when presented with the word ‘BLUE’ printed in blue ink, the correct answer is ‘blue’ on the response controller. Participants can respond quickly because the word and the font colour match.

Incongruent: In this condition, the colour name and the colour of the font differ. For example, the word ‘BLUE’ will be presented in red ink, and the correct answer will depend on inhibiting an automated response (Stroop 1935).

Following 10 practice trials for each block, for 48 trials, participants were asked to identify to the written word meaning and for 48 trials to respond to the colour of the font. Half of all trials were word-font congruent or neutral, and the other half of trials were incongruent. A new stimulus was presented 1000 ms following a response. The dependent variables of interest were reaction time and accuracy.

#### The Rapid Serial Visual Presentation paradigm

**Fig. 2:**
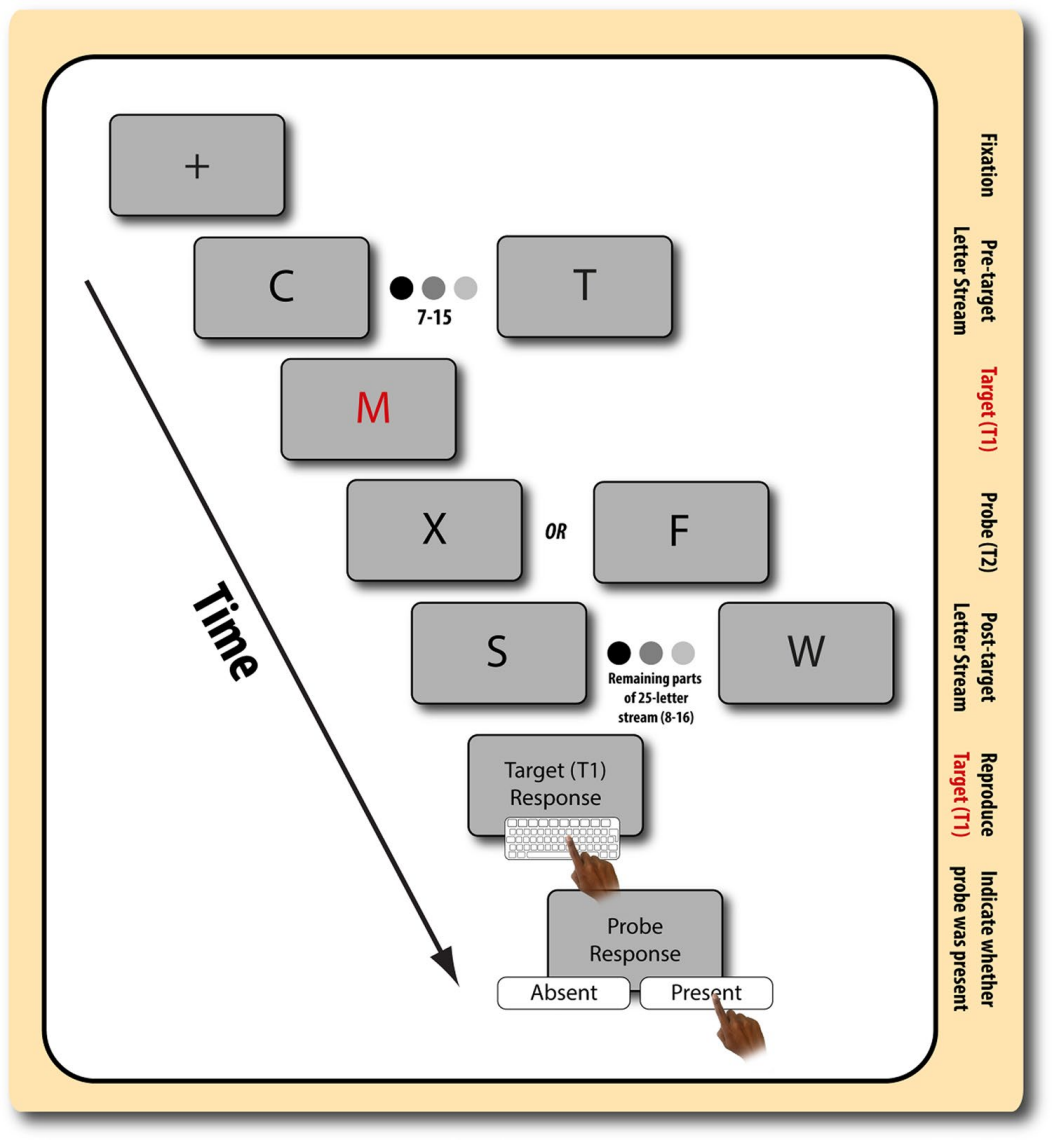
A visual representation of the RSVP trial sequence. Prior to the start of each block, participants were informed about whether they had to perform the single or dual task condition. In the single task condition, participants just had to report whether the letter ‘X’ was present in the subsequent display. In contrast, in the dual task, participants had to report both the presence/absence of the letter ‘X’ (T2) *and* the identity of the letter presented in red (T1). Each block began with a (1) fixation point to prime participant. Then, (2) a sequence of letters (varied between 7 and 15) were displayed on the screen prior to T1. (3) Target (T1) letter in presented in red. (4) A further a sequence of letters is presented, in which X was present in 50% of trials. In the dual task condition, participant inputs the red target letter on the keyboard (dual task only). (5) Then, participant inputs whether the letter X (T2) was present

RSVP involves presenting a stream of randomly chosen letters presented rapidly in succession, at the centre of the screen (Fig. 2). Each letter was presented for 131 ms with an inter stimulus interval of 49 ms equating to a presentation rate of 5.6 letters per second in keeping with recently published paradigms (Husain et al. 1997). Each RSVP stream was 25 letters long. All letters were black except the target letter (T1), which was red. The background throughout the sequence was a uniform grey. Each trial began with a black fixation cross lasting 500 ms. Prior to T1, the number of letters presented randomly varied between 7 and 15. T1 could be any letter except for ‘X’. The second target letter (T2) was a black ‘X’, randomly present in only 50% of trials. The T2 (letter X) was never presented before T1 (red letter) and no letter appeared twice within a single RSVP stream.

In the control block (single target trials), participants were requested to report the presence or absence of T2 only whereas in the testing block (dual target trials), participants were requested to identify T1 (by typing in the letter using the keyboard) followed by reporting the presence or absence of T2. T2 onset could occur after 180 ms, 360 ms, 540 ms, 720 ms, 900 ms, 1080 ms or 1260 ms. Reports of both targets were requested after the stimulus stream terminated. T2 was presented 3 times as each T2 time intervals, yielding a total of 21 T2 present and 21 T2 absent dual target trials. Participants completed 5 practice trials before each testing block. The dependent variables were response accuracy of T2 identification in the control trials and accuracy of identification of both T1 and T2 in the test trials, at each of the time intervals. In addition, we assessed speed of response for correctly identified T2 trials.

### Statistical analysis section

Using data from a similarly designed study (Brunye et al. 2010), we calculated with conventional formula (Kadam and Bhalerao 2010) that a study power of 80% would require a sample size of approximately 16 participants to demonstrate an effect.

Data were analysed using mixed ANOVAs in JASP 0.14.1 (JASP Team 2020), with the repeated and between-subjects variables indicated for each respective analysis. The threshold for statistical significance was set at the conventional level (*a* = 0.05) and appropriate estimates of effects size are also provided (e.g. *η*^2^_*p*_ for ANOVAs). Corrections applied for violations of sphericity were done using Greenhouse–Geisser method. Where found, the direction of significant interactions is uncovered using simple main effects analyses.

## Results

### Minimal effects of caffeine abstention on attention

Requiring participants to abstain from caffeine for 7 days was not found to be robustly associated with changes in attentional functioning. Rather, performance was generally found to increase from day 1 to day 7, consistent with the practice effects on the tasks.

### Caffeine improves basic response selection irrespective of disease status

**Fig. 3:**
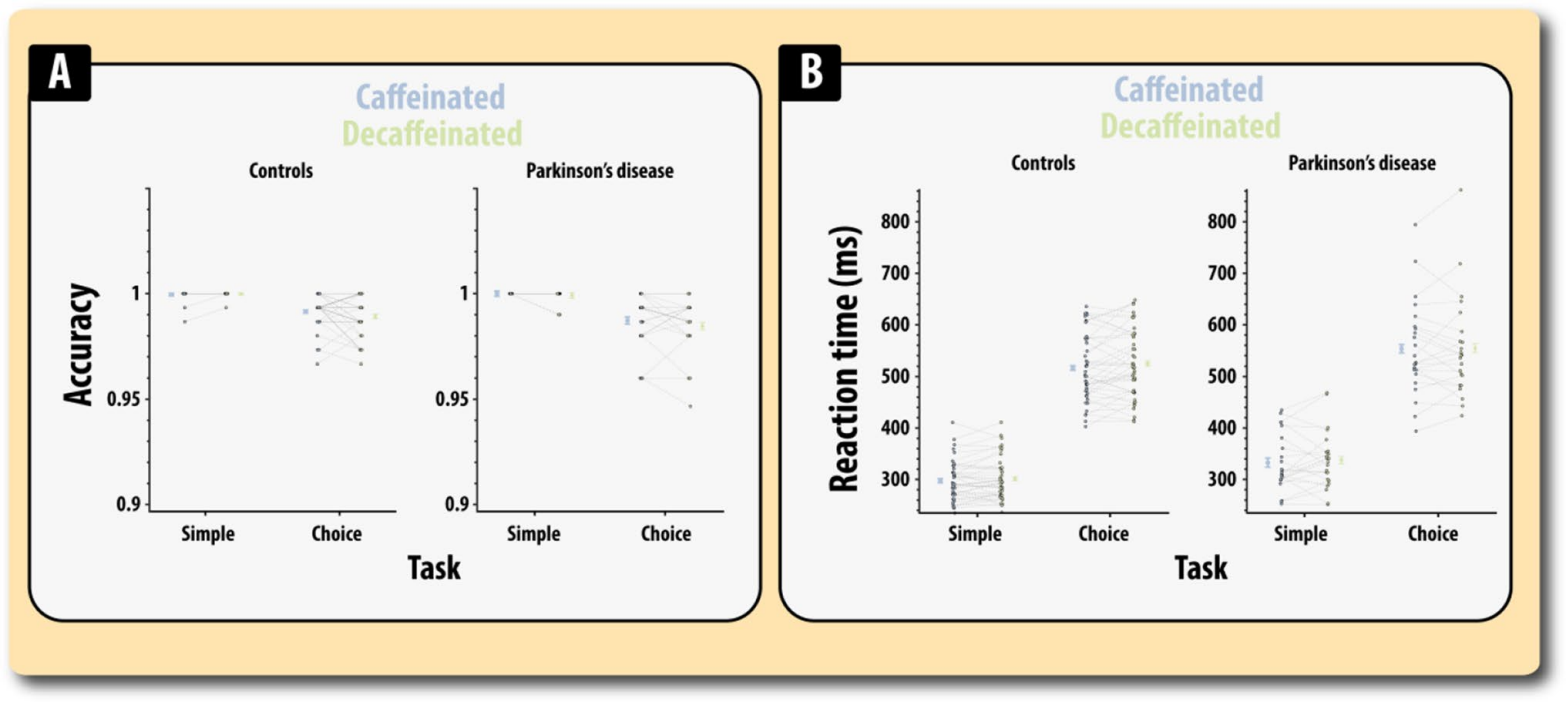
Accuracy (**A**) and reaction time (**B**) on the simple reaction time (SRT) and choice reaction time (CRT) tasks split according to disease status and caffeine status. Errors reflect the standard errors of mean

On inspection of the accuracy data for this very simple task, four extreme outliers (> 3SD below the mean) were removed from further analysis (1 healthy controls and 3 PD patients). Firstly, we examined the differential effect that caffeine and disease status had on accuracy on the choice response task (errors/mistrials in the simple reaction time were too few to enable analysis, but are shown in Fig. 3A; Table S1). Firstly, for accuracy, we performed a mixed ANOVA with drug status (caffeinated or decaffeinated) as a within-subject factor and disease status (healthy older adults, PD) as between-subject variable.

A significant main effect of drug revealed that errors on the choice reaction time task were lower in the caffeinated compared to decaffeinated state (*F*(1,64) = 4.00, *MSE* = 0.00004, *p* = 0.049, *η*^2^_*p*_ = 0.058). Thus, caffeine appeared to selectively improve the accuracy on the choice reaction time, i.e. when responses had to be mapped according to visual stimuli. There was a trend for PD patients to make more errors than controls on the choice reaction time task (*F*(1,64) = 3.43, *MSE* = 0.00001, *p* = 0.068, *η*^2^_*p*_ = 0.050). There was no evidence disease modulated the effect of drug (*F* < 1).

For response latencies (correct trials only), there was, as expected, a significant main effect of task (*F*(1,64) = 928.81, MSE = 3127. *p* < 0.0001, *η*^2^_*p*_ = 0.93) with responses being longer in the choice reaction time task compared to the simple reaction time task (Fig. 3B; Table S2). Though numerically faster on caffeine compared to placebo, there was no significant main effect of drug on response latency (*F*(1,64) = 1.72, *MSE* = 670, *p* = 0.19, *η*^2^_*p*_=0.026). Responses were significantly slower in patients (*F*(1,64) = 5.90, *MSE* = 11,967, *p* = 0.018, *η*^2^_*p*_=0.084). Thus, there was no evidence that caffeine affected response latencies. None of the other effects was significant (*F*’s < 1). Control analyses on accuracy and response latencies looking at modulatory role of day 7 performance and session order did not substantively alter the above analyses.

### Caffeine improves overall performance on the Stroop task

Inspecting accuracy across this experiment, 5 participants (3 healthy control and 2 PD patients) were removed from the analysis due to being extreme outliers (> 3SD below the mean). Firstly, we examined accuracy and response latencies (correct responses) using the same statistical model to that used for the SRT/CRT task. A mixed ANOVA with task (incongruent, congruent) and drug (caffeine, decaffeinated) as within-factors factors and disease (healthy controls, PD) as a between-subject factor was used to examine accuracy.

**Fig. 4:**
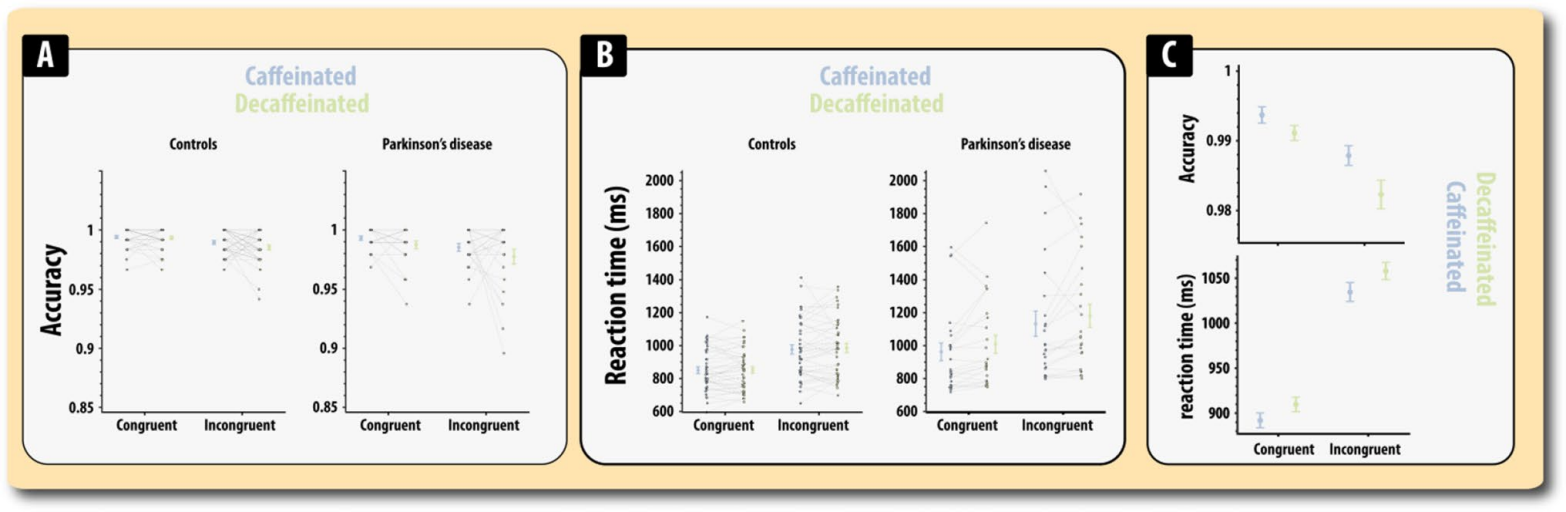
Performance on congruent and incongruent trials in the Stroop task according to caffeine and disease status. **A** Accuracy. **B** Reaction time. **C** Micrograph showing the effects of caffeine and disease on accuracy and reaction time. Errors bars reflect the standard error of the mean

As expected, accuracy was significantly higher in the congruent than the incongruent condition (*F*(1,63) = 20.12, *MSE* = 0.00017, *p* < 0.0001, *η*^2^_*p*_ = 0.242; Fig. 4A; Table S3). Accuracy was also significantly higher in the caffeinated compared to the decaffeinated state (main effect of drug; *F*(1,63) = 5.89, *MSE* = 0.00022, *p* = 0.018, *η*^2^_*p*_ = 0.086). There was also a significant main effect of disease with patients making more errors than controls (*F*(1,63) = 5.64, *MSE* = 0.00023, *p* = 0.02, *η*^2^_*p*_ = 0.082). The effect of drug did not significantly vary according to disease (*F*(1,63) = 1.20, *MSE* = 0.00022, *p* = 0.27, *η*^2^_*p*_ = 0.019) or task (*F* < 1). There was no significant main effect of disease and none of the other effects was significant (*F*’s > 1). Thus, caffeine improved accuracy on both the congruent and incongruent trials in the Stroop task suggesting the drug improves response selection — as there were multiple possible responses during both the neutral and incongruent conditions.

Using the same statistical model to evaluate response latency (correct responses only) revealed the prototypical exacerbation of response latencies in the incongruent compared to the congruent condition (*F*(1,63) = 147.80, *MSE* = 9331, *p* < 0.0001, *η*^2^_*p*_ = 0.70; Fig. 4B; Table S4). Response times were significantly quicker after administration of caffeine compared to the decaffeinated state (*F*(1,63) = 4.02, *MSE* = 10,202, *p* = 0.049, *η*^2^_*p*_ = 0.06). Drug effects did not vary by task (*F* < 1).

Patients were significantly slower than controls (*F*(1,63) = 7.79, *MSE* = 185,854, *p* = 0.007, *η*^2^_*p*_ = 0.11), but this effect did not vary according to task (disease × task interaction; *F*(1,63) = 2.94, MSE = 9331, *p* = 0.09, *η*^2^_*p*_ = 0.043). There was also no disease × drug interaction; *F*(1,63) = 2.67, *MSE* = 10,202, *p* = 0.10, *η*^2^_*p*_ = 0.041). None of the other effects was significant (*F*’s < 1). Thus, PD patients showed evidence for reaction times being speeded by drug administration, but this effect was absent in controls.

As with the CRT data, we conducted control analyses on accuracy and response latencies looking at modulatory role of day 7 performance and session order. Inclusion of these did not substantively alter the above analyses (Supplementary Information).

Cumulatively, these results show that caffeine can improve the accuracy and speed of response selection in both patients and controls.

### Caffeine differentially modulates accuracy according to task demands

Turning to the Rapid Serial Visual Presentation task, 5 participants (2 PD patients and 3 controls) were removed from the analysis of the active sessions due to incomplete data or difficulty completing the task.

A mixed ANOVA on total accuracy was performed with drug (caffeine vs. decaffeinated), task (single, dual) and AB interval (180 ms, 360 ms, 540 ms, 720 ms, 900 ms, 1080 ms or 1260 ms) as within-subject factors and disease group (PD or control) as a between-subject factor.

**Fig. 5:**
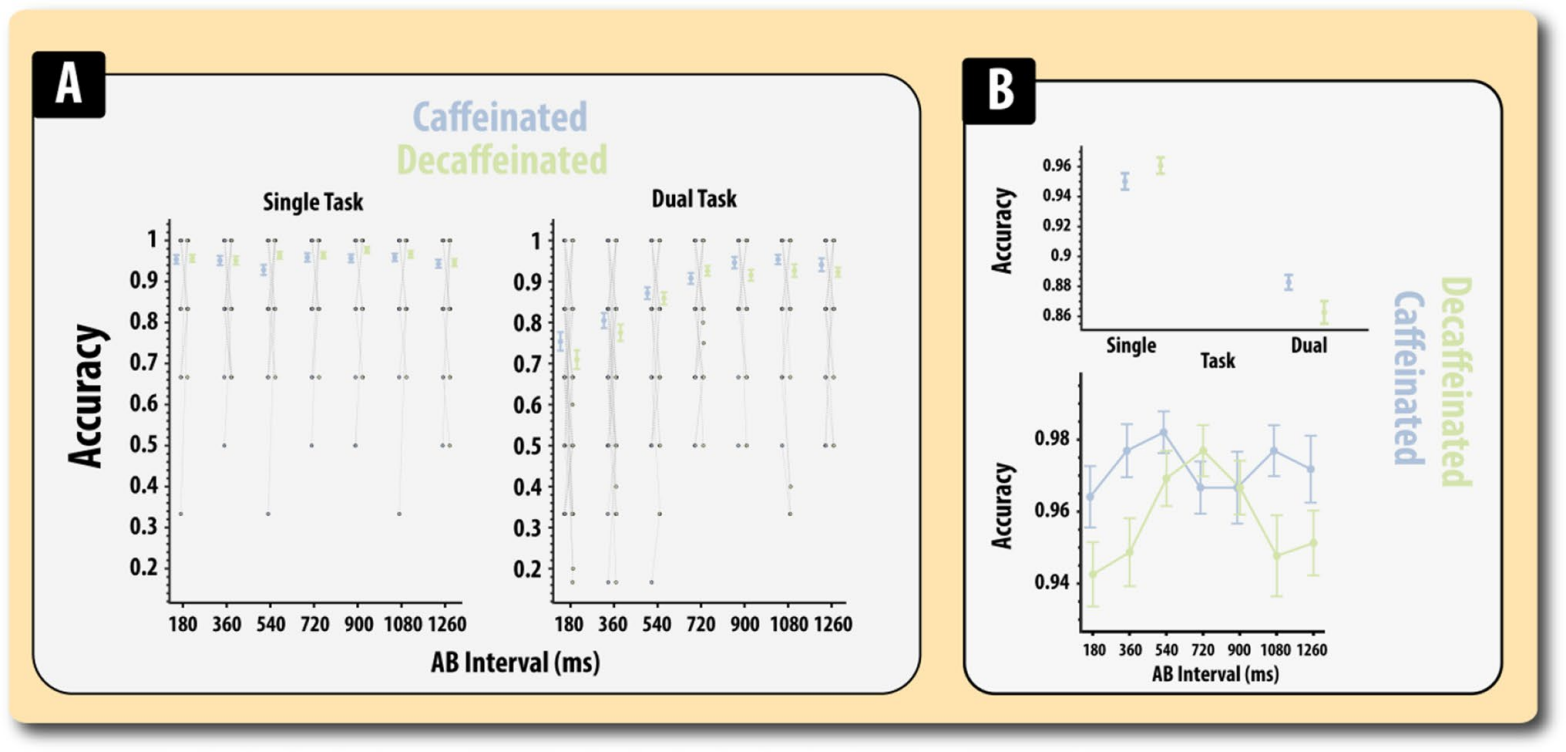
Accuracy on the single and dual task conditions in the RSVP according to caffeine status. **A** Accuracy. **B** Micrograph showing the effects of caffeine on accuracy. Top, overall accuracy, bottom (performance on target detection, T1, red letter). Errors bars reflect the standard error of the mean

As expected, the known task-related behavioural patterns were present on the caffeine and placebo days (Fig. 5A; Table S5). There was a marked decrease in accuracy for performing the dual compared to single task condition (*F*(1,63) = 76.92, *MSE* = 0.039, *p* < 0.0001, *η*^2^_*p*_ = 0.55). In addition, accuracy was generally lower for shorter compared to longer AB intervals (*F*(2.72, 245) = 27.91, *MSE* = 0.699, *p* < 0.0001, *η*^2^_*p*_ = 0.30). In line with the known dependence on the temporal dynamics of the attentional blink, there was a significant interaction between task type and AB interval (*F*(2.4, 267) = 32.53, *MSE* = 0.56, *p* < 0.0001, *η*^2^_*p*_ = 0.34). This was due to there being significantly lower accuracy (all *F*’s < 1) in the dual condition than the single condition for all AB intervals up to 1080 ms. Thus, overall, in the sample, there was no significant impairment in attention when there was a period of 1080 between T2 and T1.

With regard to the modulatory effects of drug, there was no significant main effect of caffeine on overall performance (*F* < 1). Thus, there was no overall benefit in performance from having taken caffeine. However, the effects on performance were found to significantly depend on whether the single or dual task was being performed (*F*(1,63) = 7.74, *MSE* = 0.018, *p* = 0.007, *η*^2^_*p*_=0.11). Simple main effects analysis revealed that caffeine significantly *improved* performance on the dual task condition (*F*(1,63) = 4.55, *p* = 0.037). This improvement in dual task performance co-occurred with a significant impairment in single task performance (*F*(1,63) = 4.22, *p* = 0.044). Caffeine did not significantly interact with any other experimental variable (*F*’s < 1). There was no evidence that PD patients were impaired on this task compared to controls and disease status did not significantly interact with any other variable (*F*’s < 1).

Control analyses conduct to examine the modulatory role of day 7 performance and session order on accuracy did not substantively alter the above analyses (Supplementary Information).

Cumulatively, therefore, it appears that caffeine, irrespective of disease state, improved performance when high demands were placed on the attentional system (dual task), but impaired performance when demands were low (single task).

### Caffeine improves dual task performance by aiding T1 and not T2 identification

The above analysis identified that caffeine improved performance on the dual task condition, but it does not illuminate the mechanisms through which caffeine exerts these beneficial effects. Here, we try to isolate the specific cognitive component caffeine is affecting by analysing the pattern of errors the drug produces. The difficulty of the dual task stems from the requirement to identify both T1 (correct identification of red target letter) and T2 (correct identification of presence of an X following T1). Thus, one or both of these processes may go awry during a trial, which would indicate separate deficits in attention. To examine whether this is the case, we tested the effect of caffeine on T1 verses T2 identification accuracy. To this end, we performed separate mixed ANOVA on T1 and T2 identification accuracy (dual task only) with drug (caffeine, decaffeinated) and condition (1…*n*) as within-subject factors and disease as a between-subject variable.

For the T1 identification ANOVA, there was a significant main effect of drug (*F*(1,63) = 4.30, *MSE* = 0.010, *p* = 0.042, *η*^2^_*p*_ = 0.06; Fig. 5B) in the direction of caffeine improving accuracy. There was no significant main effect of condition (*F*(6,378) = 1.40, *MSE* = 0.005, *p* = *0.26*, *η*^2^_*p*_=0.02) and no significant interaction between drug and condition (*F*(6,378) = , *MSE* = 0.004, *p* = *0.13*, *η*^2^_*p*_=0.025). None of the other effects was significant (*F* < 1).

For the T2 identification ANOVA, there was the typical decrement in accuracy with shorter AB intervals (*F*(3.1,197) = 42.07, *MSE* = 0.036, *p* < *0.0001*, *η*^2^_*p*_=0.40). The effect of AB interval was not significantly modulated by caffeine administration (*F* < 1) or disease (*F*(3.13,197) = 1.06, *MSE* = 0.036, *p* = 0.36, *η*^2^_*p*_ = 0.010), and there was no three interaction between AB interval, drug and group (*F*(4.2,266) = 1.58, *MSE* = 0.016, *p* = 0.17, *η*^2^_*p*_ = 0.02). There were no significant main effects of drug or disease (*F*’s < 1).

In summary, therefore, caffeine did not show any evidence of improving T2 identification, but there was evidence that T1 identification is bolstered by caffeine administration.

### Disease impairs the efficacy of attention according to task type

A complimentary measure of the efficacy of attention to accuracy is the time taken to correctly indicate the presence or absence of the probe (T2, the second item participants have to report). In addition, it is important to know whether any improvements in accuracy have come at the expense of prolonged response latency (i.e. a speed accuracy trade off). Accordingly, we assessed the response latency to the probe (T2) item in a mixed ANOVA with drug (caffeine, decaffeinated), task (single, dual) and AB interval (180…*n*) as within-subject factors and disease group as a between-subject factor. Only correctly performed trial was included in the analysis.

**Fig. 6:**
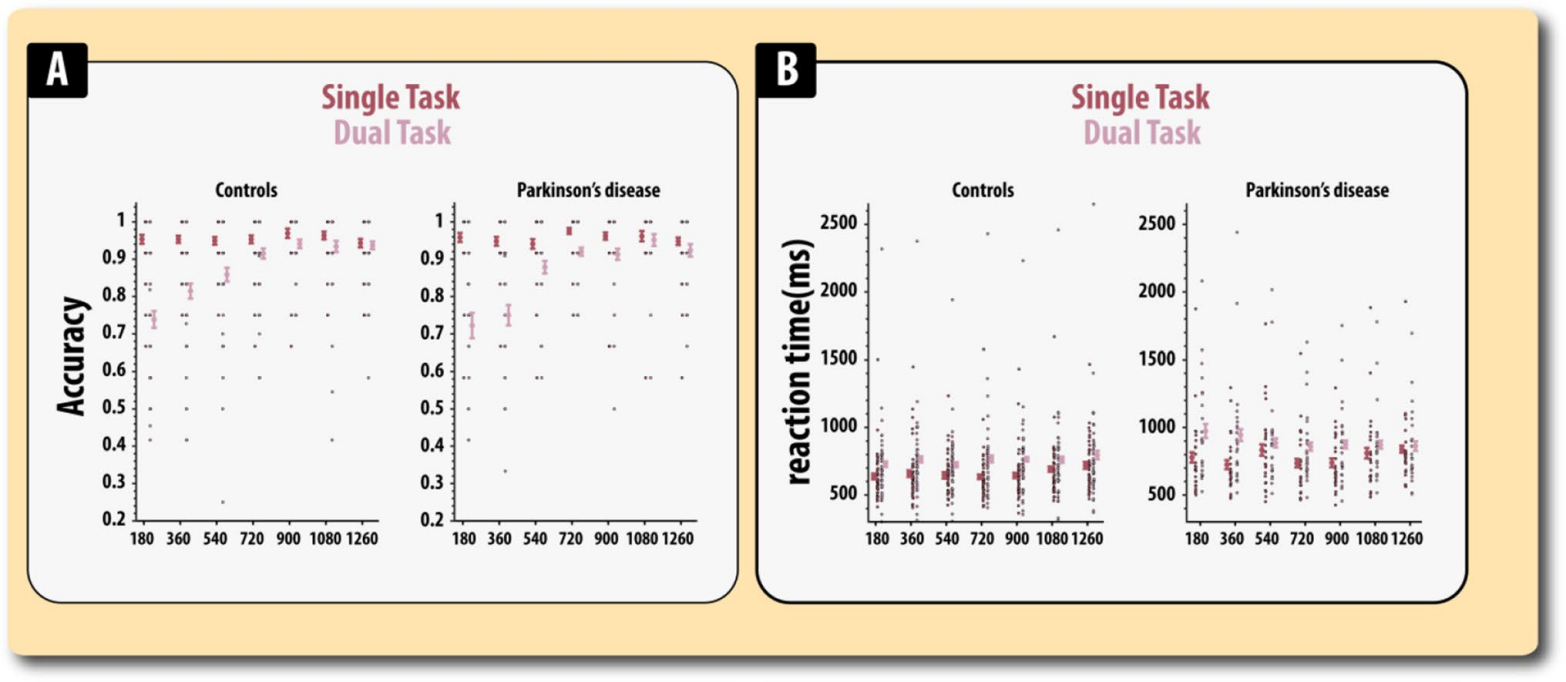
Response latencies (correct trials only) when indicating the presence of absence of the probe (T2) item according to task (single, dual) and disease status. **A** Accuracy. **B** Reaction time. Errors bars reflect the standard error of the mean

As expected, participants were significantly slower in the dual task compared to the single task (*F*(1,63) = 13.47, *MSE* = 23,652, *p* < *0.0001*, *η*^2^_*p*_=0.18; Fig. 6B; Table S6) and reaction times significantly varied according to AB interval (*F*(4.9,309) = 3.3, *MSE* = 28,929, *p* = 0.007, *η*^2^_*p*_=0.05). There was no significant main effect of drug (*F* < 1), interaction between drug and task (*F*(1,63) = 1.992, *MSE* = 96,063, *p* = *0.16*, *η*^2^_*p*_=0.03), drug and AB interval (*F*(5.48,345) = 1.17, *MSE* = 22,754, *p* = *0.32*, *η*^2^_*p*_=0.018) or significant three-way interaction between drug, task and AB interval (*F*(5.16,325) = 1.45, *MSE* = 26,864, *p* = *0.20*, *η*^2^_*p*_=0.02).

PD patients were found to be significantly slower to respond than health older adults (*F*(1,63) = , *MSE* = 1,445,000, *p* = 0.032, *η*^2^_*p*_=0.07) — as expected. However, patients’ impairment was found to significantly vary according to AB interval (*F*(4.9,309) = 4.28, *MSE* = 28,929, *p* = *0.0009*, *η*^2^ = 0.06). This two-way interaction was itself superseded by a three-way interaction between task, AB interval and disease group (*F*(4.98,313.5) = 2.40, *MSE* = 33,224, *p* = *0.037*, *η*^2^_*p*_=0.04). To decompose this interaction, we subtracted the response latencies in the single condition from the dual condition (dual minus single) at each AB interval for patients and healthy adults to produce a task cost score. Within groups, the task cost score was found to significantly vary with AB interval for PD patients (*F*(4.1,96) = 2.83, *MSE* = 135,563, *p* = 0.027, *η*^2^_*p*_=0.11), but there was no evidence for this in healthy controls (*F*(4.1,172) = 1.52, *MSE* = 45,244, *p* = 0.192, *η*^2^_*p*_ = 0.04). The comparison between groups across different AB intervals revealed that although numerical differences were larger for the shorter AB intervals, none of these differences was significant (all *p*’s > 0.16). The aforementioned differences between patients and controls did not significantly vary according to drug state (*F*(5.16,325.4) = 1.95, *MSE* = 26,864, *p* = *0.08*, *η*^2^_*p*_=0.03). None of the other interactions was significant. In summary, analysis of the reaction time data revealed evidence for an impairment in PD patients, which was most prominent for the short AB intervals.

Again, control analyses conduct to examine the modulatory role of day 7 performance and session order on response latencies did not substantively alter the above analyses (Supplementary Information).

## Discussion

Here we demonstrate dissociable effects of caffeine in older people with and without PD depended on task requirements and the type of attentional demands that needed to be overcome. We employed a more rigorous testing procedure to ensure adequate time for participants to be fully withdrawn from caffeine before being randomised to caffeine or placebo.

There was no evidence that caffeine affected attention in a unitary manner, across all attentional networks (Petersen and Posner 2012). Rather, the effect of caffeine on cognitive performance depended greatly upon the specific task requirements. Caffeine improves accuracy on the choice reaction time task. A similar benefit — which likely shares the same cognitive foundations — was found in the Stroop task. Caffeine improved accuracy of response selection in both the congruent and incongruent conditions.

Consistent with effects of caffeine on dual task performance (van Duinen et al. 2005), caffeine increased performance accuracy in the dual task condition of the attentional blink paradigm but paradoxically impaired performance in the single task condition. Analysis of the pattern of errors participants made on this task revealed that the improvement in the dual task was due to enhanced recall of the target red letter (T1) and not the presence of absence of the second stimulus (which was always an X).

### Caffeine improves response selection

Caffeine is frequently taken to improve performance and may have utility as a cognitive enhancer in neurodegenerative conditions, such as PD but providing robust evidence for the cognitive functions that caffeine does, and does not, affect has been lacking. Utilising our full withdrawal design this study provides important new insights into the selective effects of caffeine on cognitive functioning in health and disease.

Only a handful of published randomised controlled trials have tested the acute attentional effects of caffeine following a withdrawal period of 4 days or longer whilst the majority of published trials typically use a withdrawal period of less than 48 h (Judelson et al. 2005; Kamimori et al. 2015; Rogers et al. 2005; Smith et al. 2013). Consistent with some of these findings, in this study, the clearest evidence for cognitive gains after caffeine administration was found in the accuracy scores for the choice reaction time task (Fig. 3) and overall accuracy in the Stroop task (Fig. 4). Evidence from electrophysiological studies have reliably shown that caffeine increases ERPs that are related to the selective processing of relevant information (Lorist et al. 1994, 1995; Ruijter et al. 2000) suggesting that caffeine may lead to enhanced performance when relevant information must be selected over irrelevant information as is required during incongruent trials in the Stroop task, i.e. the font colour needs to be responding to whilst ignoring the colour name. However, demonstrating a selective benefit for caffeine on errors in the incongruent trials has proved controversial with multiple findings being reported depending on the nature of the paradigm and the pharmacological procedure (Kenemans et al. 1999). In this study, there was no evidence that caffeine had selective behavioural effects on accuracy in the congruent or incongruent conditions, rather errors appeared to be reduced in both the congruent and incongruent conditions (Fig. 4). This suggests that this effect is not driven by caffeine enhancing the activity of networks that enable selective attention, but something more general. An alternative hypothesis for these effects is that caffeine improved response selection, the ability to correctly couple a perceptual feature (colour) to a specific response (as was clearly demanded in this study’s version of the Stroop). This explanation would align well with the finding that caffeine improved accuracy on the choice reaction time task. Also, it seems unlikely that this effect is due to some non-specific effect such as generic changes in attentional lapses, motivation, vigilance or other deficits in sustained attention as contrasting effects were found in the RSVP task.

### Caffeine only improves response latency on the Stroop task

One of the most prominent findings in the literature on the cognitive effects of caffeine is that it can speed up motor responses on choice reaction time tasks (e.g. Liberman, effects of low dose caffeine on human performance and mood). We did not observe a significant quickening of response on caffeine on the serial or choice reaction time tasks. One obvious explanation for this is that caffeine is exert different effects across the attentional networks that support these tasks (Petersen and Posner 2012), gains on the Stroop task are supported by caffeine modulating the executive network and effects on the choice reaction time are due to effects on the alerting network. Alternatively, this discrepancy could be due attributed to the age of the sample population and disease effects, who due to normal or abnormal ageing, respectively, lack the ability to obtain this effect after caffeine. However, this seems unlikely given that reactions were found to be speeded up in the Stroop task after caffeine administration. It is possible though that there is some task-specific effect of our protocol (e.g. multiple testing sessions affecting arousal or novelty) that prevented us from observing significant effects of caffeine on response latency in the CRT. Future studies should seek to implement designs that allow these different explanations to be assessed. Specifically, the inclusion of a younger sample of participants would also clarify the extent to which the effects observed here were due to ageing or the protocol.

### Caffeine differentially modulates accuracy according to task demands

There was no evidence that caffeine appreciably modulated the nature of the attentional blink in patients or controls (Fig. 5). Rather, caffeine was found to have negative or positive effects according to the whether the single or dual task was being performed, i.e. enhanced performance on dual task conditions co-occurred with relatively impaired performance on the single task condition.

Ostensibly, this finding of impaired or improved performance after drug administration is entirely in line with the results from other putative cognitive enhancers (Fallon et al. 2016a). In keeping with the Yerkes-Dodson theorem, performance with respect to the level arousal, or redolent form of neurochemical stimulation, is proposed to follow an inverted U-shaped curve (Anderson 1994). Optimum performance for a given task does not occur when arousal is maximal, but rather when intermediate states have been achieved. Different tasks are seen to require different optima, such that improving performance on one task may impair another (Roshan Cools & D’Esposito 2011; Mattay et al. 2003). Within this framework, it could be argued that the single and the dual tasks have different optimum levels of arousal, or neurostimulation, associated with them and therefore improved performance on the dual task comes at the expense of the impaired performance on the single task.

It would be tempting to argue that caffeine improves performance on the high demand task (dual) but impairs performance on the low demand task (single). However, this framing would have trouble accommodating the other low-demand tasks in this study, such as the choice reaction time task, which caffeine was found to improve. It is likely, therefore, that the answer may reside in the unique attentional requirements of the RSVP task.

A potentially important clue in understanding this effect is the finding that caffeine improves dual task performance by aiding T1 (red letter) and not T2 (presence or absence of an X) identification. During the dual task condition, caffeine improved performance by augmenting T1 identification with no effect on T2 identification. This runs contra to most models of the attentional blink, which often argue that sensory processing of T1 will inhibit processing of T2; for a detailed review of the attentional blink, see Dux and Marois (2009). However, our results demonstrate enhanced performance on the dual task condition due to improved identification of T1 rather than T2, suggesting that caffeine is primarily acting to enhance the processing of T1.

One hypothesis to explain this result is that caffeine serves to boost processing of salient information such as T1 irrespective of whether the reporting of this letter is required during the current block, i.e. in both the single and the dual task conditions. The enhanced processing of T1 would result in superior performance on the dual task condition, but impaired performance on the single task condition. Mechanistically, such an effect could be engineered by caffeine-mediated dopaminergic effects in the basal ganglia.

Dopamine D2-receptor expressing neurons in the basal ganglia have been found to be involved in regulating the attentional blink. Specifically, a position emission tomography (PET) study has found that individuals with higher levels of D2 receptors in the striatum have a larger attentional blink (Slagter et al. 2012), i.e. higher levels of D2 receptors are associated with an increase in the failure to identify T2 after correctly identifying T1. Administration of dopamine to PD patients can decrease the size of the attentional blink in some patients (Slagter et al. 2016). An unresolved question is the extent to which the role of the D2 receptor in the attentional blink is due to the gating of items in working memory. A large corpus of evidence points for a role of the D2 dopamine receptor in preventing unwanted information from entering working memory (Frank; Cools, Chatham) (Broadway et al. 2018; Chatham & Badre 2015; Fallon et al. 2019; Frank & O’Reilly 2006) with a prominent role for the dopamine D2-receptor expressing neurons in the indirect pathway in filtering out irrelevant (non-needed) information. Previously, administration of a D2 agonist, cabergoline, was found to causes an undifferentiated amplification of sensory information into working memory with the consequence that the entry of isolated information into working memory was enhanced but improved when presented in the context of multiple items (Fallon et al. 2016b). Such a mechanism may also be at work here. However, further work, perhaps involving pharmacological manipulations, will be needed in order to verify this account.

### Little evidence that caffeine has different effects in healthy controls and PD patients

One of the main hypotheses we wanted to explore was whether the effect of caffeine varied according between health control and participants with Parkinson’s disease. Studies have suggested that there is a lower density of adenosine receptors in the dorsal striatum in PD compared to controls (Hurley et al. 2000) and that these receptors are co-localised on D2-expressing neurons in the indirect pathway. Such findings would suggest that PD patients may differ in their cognitive response to caffeine. However, across all times, this was not found to be the case.

### The effect of prolonged caffeine abstinence on attention is limited

With the exception of PD patients on the CRT, testing participants following a 1-week caffeine abstention was not associated with impaired performance compared to baseline testing whilst on habitual caffeine consumption. Rather, as expected, testing participants after abstinence was associated with improved performance, mostly likely due to practice effects. Most studies to date were conducted during the acute withdrawal period and our results do not detract from the *caffeine withdrawal reversal hypothesis* (James and Rogers 2005, Yeomans et al. 2002, Bruce et al. 1991, James 1998). Instead, these results confirm an abstention period of a week is long enough to pass beyond withdrawal, avoiding the associated negative attentional effects. Any future research examining the acute cognitive effects of caffeine should ensure a similar duration of abstention as a minimum. Future research is required to ascertain whether the effects of caffeine abstention found here are genuinely due to the removing caffeine from daily life or are driven by practice effects.

### Limitations

The optimal caffeine dose to enhance attention is not known. We opted for a moderate dose of 100 mg caffeine as the intervention, which is greater than found in foodstuffs but smaller than any other trial which fully withdrew participants prior to testing (Judelson et al. 2005; Kamimori et al. 2015; Rogers et al. 2005; Smith et al. 2013). Caffeine doses as low as 20 mg and as high as 800 mg/day have been trialled but few studies have performed head-to-head comparisons of the effect of different doses on the same cohort using the same attention tests (Kamimori et al. 2015; Lieberman et al. 1987). It is perhaps because of the assumption that the greater the caffeine dose, the greater the attentional enhancement. This is in juxtaposition to the Yerkes-Dodson law where it is conceivable, a low or moderate dose may be more effective than high-dose caffeine and that the optimum dose is task demand specific.

The effect of caffeine was modest and whilst this could be attributed to the dose, it may be that it is simple a weaker attentional enhancer than other medications, such as amphetamine or modafinil. However, it will be important for future studies to directly compare the cognitive enhancing effects of caffeine to those obtained with these other cognitive enhancers. The almost ubiquitous use of caffeine means that whilst the individual effect might be small, at a population level, the effect is much larger. We have demonstrated an effect when using neuropsychological paradigms in a controlled environment and the next step is to establish whether this translates to a real-world benefit.

Moreover, assessing whether these potentially small acute effects of caffeine translate into a larger clinical benefit will require longer trials and longer follow-up periods. These are necessary to establish whether caffeine could be a viable cognitive enhancer in neurological populations.

The purpose of the manuscript was to compare the effect of caffeine across different tests of attention to evaluate whether caffeine has the same, or different, effects across different attentional tasks. Thus, in seeking to elucidate the effects of caffeine on different attentional networks, we have performed numerous statistical comparisons. This could lead to the suggestion that some of the effects observed here are false positives. However, the effects of caffeine on the Stroop task are not consistent with this view. On the Stroop task, we found improvements in both response latency and accuracy, suggesting the cognitive components that contribute to this task were generally augmented. Again, further trials in larger samples of patients will be needed to confirm the above findings.

A limitation of this study is that we did not collect data on various physiological substrates known to affect caffeine metabolism, e.g. CYP1A2 and ADORA2A genotype, drugs modulating CYP1A2 activity. Additional testing for ADORA2A gene polymorphism could predict the sensitivity of an individual to the antagonising effects of caffeine on adenosine receptors. The CYP1A2 gene is responsible for the main hepatic enzyme which metabolises caffeine, with several single nucleoside polymorphisms associated with increased caffeine clearance (Landolt 2012; Djordjevic et al. 2010; Nehlig 2018). Future studies should seek to stratify participants by these variables in order to ascertain discrete cognitive effects.

### Conclusion

A body of research spanning over a century has championed caffeine as a panacea for impaired attention. In contrast to the majority of published data, this study (i) applied an appropriate caffeine withdrawal period of 1 week prior to testing; (ii) employed a battery of neuropsychological tests to comprehensively assess visual attention and (iii) demonstrated a differential improvement in selective visual attention dependent on task difficulty, following acute caffeine administration. There were no group differences between PD and aged matched healthy elderly participants. We demonstrated beneficial effects on the Stroop task independent of withdrawal. In the RSVP task, we propose caffeine enhances the AB by either a beneficial effect on working memory or by improving the process required for discriminating between distractors from target stimuli, improving T1 target accuracy but at a cost to identifying T2. This study posits caffeine’s overall attentional effects should be considered neither exclusively positive nor detrimental but instead conditional on task demands.

## Supplementary Information

Below is the link to the electronic supplementary material.

Supplementary file1 (DOCX 319 KB)
