# Supplementary material for: Caffeine and attentional control: improved and impaired performance in healthy older adults and Parkinson’s disease according to task demands
Source: Psychopharmacology (Berl). 2022 Jan 10;239(2):605–19. doi: 10.1007/s00213-021-06054-9 (PMC8799544; doi:10.1007/s00213-021-06054-9)
Supplement: Supplementary file 1 — Supplementary file1 (DOCX 319 KB) [file 213_2021_6054_MOESM1_ESM.docx]

**Supplementary Data**

Methods


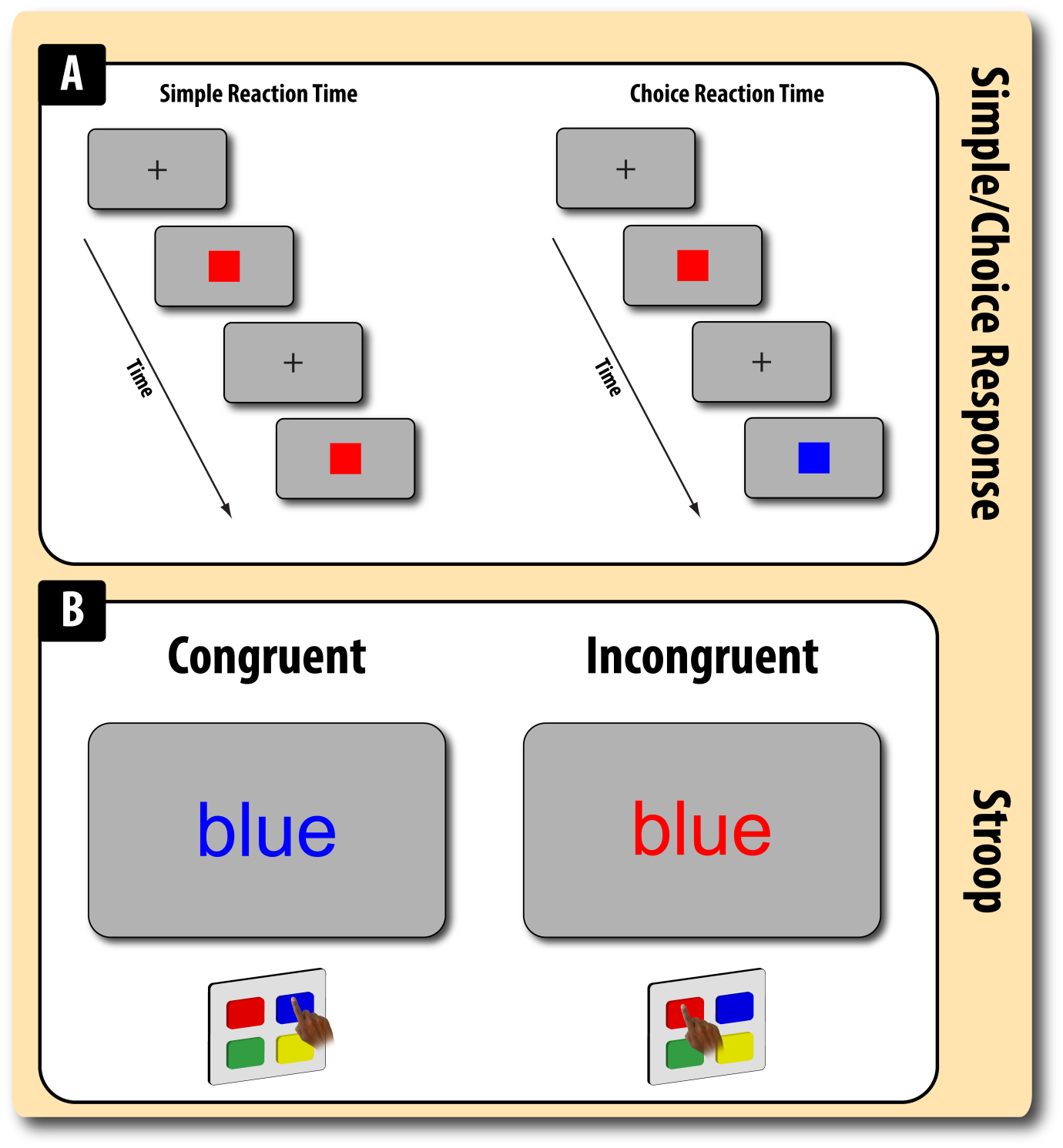


***Figure S1****. A) Simple (Left) and Choice (Right) reaction time tasks. In the Simple reaction time task participants should press a button every time a stimulus appears. In the choice reaction time task participants must press the red button for red stimuli and the blue button for blue stimuli. (B) Stroop task. Participants are presented with the name of a colour in a coloured font in the centre of the screen, and they must identify the colour of the font by pressing the corresponding button on the response box. There are two conditions a congruent condition where the font colour and the colour name are same and an incongruent condition when the font colour and the colour name are different.*

Additional Results

**Simple and Choice Reaction times**

| Drug | Task | Disease | Mean Accuracy | SD | N |
| --- | --- | --- | --- | --- | --- |
| Caff | Choice | HC | 0.99953488 | 0.00225156 | 43 |
|  |  | PD | 1.00000000 | 0.00000000 | 23 |
|  | Simple | HC | 0.99147287 | 0.00746428 | 43 |
|  |  | PD | 0.98724638 | 0.01269901 | 23 |
| Decaff | Choice | HC | 0.99984496 | 0.00101666 | 43 |
|  |  | PD | 0.99913043 | 0.00288104 | 23 |
|  | Simple | HC | 0.98914729 | 0.00965251 | 43 |
|  |  | PD | 0.98463768 | 0.01309403 | 23 |

**Table S1.** Accuracy (proportion correct) for patients and controls on the choice and simple reaction time tasks. HC= Healthy Controls. PD = Parkinson’s disease.

| Drug | Task | Disease | Mean Reaction Time | SD | N |
| --- | --- | --- | --- | --- | --- |
| Caff | Choice | HC | 516.78037276 | 65.06280585 | 43 |
|  |  | PD | 553.33960983 | 92.15781353 | 23 |
|  | Simple | HC | 297.53761458 | 38.53056544 | 43 |
|  |  | PD | 332.62313044 | 54.90161001 | 23 |
| Decaff | Choice | HC | 524.97149181 | 66.62046464 | 43 |
|  |  | PD | 554.31657998 | 97.73254407 | 23 |
|  | Simple | HC | 301.16506862 | 41.31560609 | 43 |
|  |  | PD | 337.47937550 | 56.66661278 | 23 |

**Table S2.** Response latencies (correct trials only) for patients and controls on the choice and simple reaction time tasks. HC= Healthy Controls. PD = Parkinson’s disease.

For both accuracy and response latency, we conducted some control analyses to see if correcting for pre-caffeine administration performance (Day 7 accuracy) altered the results. After adding the day 7 accuracy on both tasks as a normalised covariate, there was still a significant main effect of caffeine (F(1,63) = 4.18, MSE = .00003, p = .044, η^2^_p_ = .062). Also, adding session testing order as a between-subject variable revealed that accuracy was significantly higher in the group that were administered caffeine first and decaffeinated second, compared to the reverse order (F(1,61) = 5.02, MSE = .00005, p = .028, η^2^_p_ = .07). There was no evidence for significant drug administration order by drug interactions (*F*<1). Accounting for baseline differences in response latency (day 7) performance did not change the above results. There was no main effect of drug administration order or significant drug x order interactions (F<1).

**Stroop task**

| Drug | Task | Disease | Mean Accuracy | SD | N |
| --- | --- | --- | --- | --- | --- |
| Caff | Congruent | HC | 0.99410569 | 0.00795908 | 43 |
|  |  | PD | 0.99305556 | 0.00904286 | 23 |
|  | Incongruent | HC | 0.98943089 | 0.00986443 | 43 |
|  |  | PD | 0.98524306 | 0.01593641 | 23 |
| Decaff | Congruent | HC | 0.99329268 | 0.00858364 | 43 |
|  |  | PD | 0.98741319 | 0.01624790 | 23 |
|  | Incongruent | HC | 0.98516260 | 0.01363108 | 43 |
|  |  | PD | 0.97743056 | 0.02972846 | 23 |

**Table S3.** Accuracy (proportion correct) for patients and controls on the Stroop task. HC= Healthy Controls. PD = Parkinson’s disease.

| Drug | Task | Disease | Mean Reaction Time | SD | N |
| --- | --- | --- | --- | --- | --- |
| Caff | Congruent | HC | 856.62491960 | 129.62806876 | 43 |
|  |  | PD | 971.02192267 | 263.56373240 | 23 |
|  | Incongruent | HC | 982.76517676 | 179.65308293 | 43 |
|  |  | PD | 1146.84087557 | 375.43449443 | 23 |
| Decaff | Congruent | HC | 858.25382182 | 125.78515597 | 43 |
|  |  | PD | 1019.54261080 | 270.19275742 | 23 |
|  | Incongruent | HC | 990.93238648 | 175.43502742 | 43 |
|  |  | PD | 1197.86226489 | 342.43639300 | 23 |

**Table S4.** Response latencies (correct trials only) for patients and controls on Stroop task. HC= Healthy Controls. PD = Parkinson’s disease.

As with the SRT and CRT data, we conducted some control analyses on accuracy and response latency to examine the effect of individual differences prior to caffeine administration into our statistical model. 3 data sets were incomplete and these participants were thus excluded from the model. Here, after adding overall accuracy for the Stroop task on day 7 as a normalised covariate, there was still a significant, positive effect of caffeine administration on performance (F(1,59) = 6.60, MSE = .0002, p = .012, η^2^_p_ = .10). Adding testing session into the model did not reveal a main effect of session order or any drug by session interactions (*F*<1). Accounting for individual differences by including normalised performance on day 7 (as in the above analysis of Stroop accuracy) as a covariate did not alter significance of the main effect of drug (F(1,59) = 4.19, MSE = 10709, p .045, η^2^_p_ = .066). Adding drug administration order did not reveal any significant main effects of order (F<1), but there was a trend towards a significant interaction between drug order and the effects of drug (F(1,57) = 3.37, MSE = 10450, p =.07, η^2^_p_ = .05).

**Rapid Serial Visual Presentation task**

| Drug | Task | AB interval | Group | Mean Accuracy | SD | N |
| --- | --- | --- | --- | --- | --- | --- |
| Caff | Dual | 180 | HC | 0.77235772 | 0.19277253 | 41 |
|  |  |  | PD | 0.72222222 | 0.21234069 | 24 |
|  |  | 360 | HC | 0.81707317 | 0.19651434 | 41 |
|  |  |  | PD | 0.78472222 | 0.20547557 | 24 |
|  |  | 540 | HC | 0.87398374 | 0.18173732 | 41 |
|  |  |  | PD | 0.86805556 | 0.13882849 | 24 |
|  |  | 720 | HC | 0.89430894 | 0.15255280 | 41 |
|  |  |  | PD | 0.93055556 | 0.10897709 | 24 |
|  |  | 900 | HC | 0.95528455 | 0.07476250 | 41 |
|  |  |  | PD | 0.93055556 | 0.12925527 | 24 |
|  |  | 1080 | HC | 0.95121951 | 0.11927968 | 41 |
|  |  |  | PD | 0.95833333 | 0.08860159 | 24 |
|  |  | 1260 | HC | 0.95121951 | 0.10700404 | 41 |
|  |  |  | PD | 0.92361111 | 0.12983794 | 24 |
|  | Single | 180 | HC | 0.95528455 | 0.09877296 | 41 |
|  |  |  | PD | 0.95138889 | 0.15131602 | 24 |
|  |  | 360 | HC | 0.95121951 | 0.09312404 | 41 |
|  |  |  | PD | 0.95138889 | 0.11504218 | 24 |
|  |  | 540 | HC | 0.93902439 | 0.09686831 | 41 |
|  |  |  | PD | 0.90972222 | 0.17010345 | 24 |
|  |  | 720 | HC | 0.95121951 | 0.11330822 | 41 |
|  |  |  | PD | 0.97222222 | 0.08025724 | 24 |
|  |  | 900 | HC | 0.96341463 | 0.09510371 | 41 |
|  |  |  | PD | 0.94444444 | 0.12689783 | 24 |
|  |  | 1080 | HC | 0.96341463 | 0.07916399 | 41 |
|  |  |  | PD | 0.95138889 | 0.14311212 | 24 |
|  |  | 1260 | HC | 0.93902439 | 0.11027804 | 41 |
|  |  |  | PD | 0.95138889 | 0.09167216 | 24 |
| Decaff | Dual | 180 | HC | 0.70243902 | 0.24168279 | 41 |
|  |  |  | PD | 0.72222222 | 0.22876832 | 24 |
|  |  | 360 | HC | 0.81056911 | 0.21569513 | 41 |
|  |  |  | PD | 0.71527778 | 0.21127155 | 24 |
|  |  | 540 | HC | 0.84146341 | 0.18238855 | 41 |
|  |  |  | PD | 0.88888889 | 0.13608276 | 24 |
|  |  | 720 | HC | 0.93617886 | 0.10761960 | 41 |
|  |  |  | PD | 0.90972222 | 0.13882849 | 24 |
|  |  | 900 | HC | 0.92682927 | 0.11210598 | 41 |
|  |  |  | PD | 0.89583333 | 0.14589802 | 24 |
|  |  | 1080 | HC | 0.91626016 | 0.15725456 | 41 |
|  |  |  | PD | 0.94444444 | 0.14468579 | 24 |
|  |  | 1260 | HC | 0.92276423 | 0.12415023 | 41 |
|  |  |  | PD | 0.92361111 | 0.12983794 | 24 |
|  | Single | 180 | HC | 0.95121951 | 0.08534165 | 41 |
|  |  |  | PD | 0.96527778 | 0.06914185 | 24 |
|  |  | 360 | HC | 0.95528455 | 0.10556982 | 41 |
|  |  |  | PD | 0.94444444 | 0.09410995 | 24 |
|  |  | 540 | HC | 0.95934959 | 0.08960165 | 41 |
|  |  |  | PD | 0.97222222 | 0.06344892 | 24 |
|  |  | 720 | HC | 0.95528455 | 0.08353634 | 41 |
|  |  |  | PD | 0.97916667 | 0.05630533 | 24 |
|  |  | 900 | HC | 0.97560976 | 0.07032638 | 41 |
|  |  |  | PD | 0.97916667 | 0.07473787 | 24 |
|  |  | 1080 | HC | 0.96341463 | 0.07916399 | 41 |
|  |  |  | PD | 0.97222222 | 0.06344892 | 24 |
|  |  | 1260 | HC | 0.94715447 | 0.08691490 | 41 |
|  |  |  | PD | 0.94444444 | 0.12689783 | 24 |

**Table S5.** Accuracy (both probe and target correctly identified) for patients and controls on the RSVP task. HC= Healthy Controls. PD = Parkinson’s disease.

| Drug | Task | AB interval | Group | Mean Reaction Time | SD | N |
| --- | --- | --- | --- | --- | --- | --- |
| Caff | Dual | 180 | HC | 725.16626016 | 232.99363672 | 41 |
|  |  |  | PD | 982.11347222 | 438.21356779 | 24 |
|  |  | 360 | HC | 744.93410569 | 269.57701147 | 41 |
|  |  |  | PD | 909.57729167 | 430.47651632 | 24 |
|  |  | 540 | HC | 732.04365854 | 261.49082289 | 41 |
|  |  |  | PD | 835.84958333 | 287.66171014 | 24 |
|  |  | 720 | HC | 730.40780488 | 268.32317480 | 41 |
|  |  |  | PD | 848.97222222 | 336.79597160 | 24 |
|  |  | 900 | HC | 763.74479675 | 261.01835577 | 41 |
|  |  |  | PD | 860.12979167 | 308.11774177 | 24 |
|  |  | 1080 | HC | 738.39719512 | 313.22503231 | 41 |
|  |  |  | PD | 902.96805556 | 342.86773526 | 24 |
|  |  | 1260 | HC | 771.17715447 | 355.07098713 | 41 |
|  |  |  | PD | 829.25652778 | 259.98243010 | 24 |
|  | Single | 180 | HC | 666.40902439 | 276.43811110 | 41 |
|  |  |  | PD | 780.73256944 | 302.78057574 | 24 |
|  |  | 360 | HC | 677.25857724 | 275.69391571 | 41 |
|  |  |  | PD | 739.03833333 | 245.89877382 | 24 |
|  |  | 540 | HC | 670.75723577 | 190.80587124 | 41 |
|  |  |  | PD | 858.57715278 | 395.58753743 | 24 |
|  |  | 720 | HC | 650.26727642 | 222.99201777 | 41 |
|  |  |  | PD | 687.34729167 | 202.49391261 | 24 |
|  |  | 900 | HC | 661.08764228 | 220.46915759 | 41 |
|  |  |  | PD | 752.13791667 | 238.96880664 | 24 |
|  |  | 1080 | HC | 686.66869919 | 214.26096151 | 41 |
|  |  |  | PD | 775.11500000 | 229.14407824 | 24 |
|  |  | 1260 | HC | 727.41804878 | 222.28523483 | 41 |
|  |  |  | PD | 845.91569444 | 236.48072883 | 24 |
| Decaff | Dual | 180 | HC | 721.31028455 | 413.82880909 | 41 |
|  |  |  | PD | 973.21138889 | 395.99192398 | 24 |
|  |  | 360 | HC | 785.98674797 | 410.98830570 | 41 |
|  |  |  | PD | 980.69513889 | 465.39981098 | 24 |
|  |  | 540 | HC | 719.64239837 | 287.35119959 | 41 |
|  |  |  | PD | 926.61548611 | 416.94356399 | 24 |
|  |  | 720 | HC | 807.34439024 | 429.08666064 | 41 |
|  |  |  | PD | 864.30951389 | 275.11812547 | 24 |
|  |  | 900 | HC | 765.37853659 | 356.21431522 | 41 |
|  |  |  | PD | 891.15500000 | 353.79412813 | 24 |
|  |  | 1080 | HC | 787.29304878 | 380.42712952 | 41 |
|  |  |  | PD | 844.34347222 | 259.19709201 | 24 |
|  |  | 1260 | HC | 815.34475610 | 437.85077974 | 41 |
|  |  |  | PD | 893.65881944 | 340.86744613 | 24 |
|  | Single | 180 | HC | 605.94495935 | 122.91187390 | 41 |
|  |  |  | PD | 779.00555556 | 366.62466002 | 24 |
|  |  | 360 | HC | 638.72792683 | 216.91893826 | 41 |
|  |  |  | PD | 709.73062500 | 200.57278135 | 24 |
|  |  | 540 | HC | 618.99272358 | 160.54387403 | 41 |
|  |  |  | PD | 803.23805556 | 369.47152012 | 24 |
|  |  | 720 | HC | 617.83304878 | 190.94254848 | 41 |
|  |  |  | PD | 787.68138889 | 330.06951268 | 24 |
|  |  | 900 | HC | 626.99382114 | 180.13834454 | 41 |
|  |  |  | PD | 723.25229167 | 230.75731404 | 24 |
|  |  | 1080 | HC | 694.82857724 | 223.28394342 | 41 |
|  |  |  | PD | 834.71625000 | 436.12418123 | 24 |
|  |  | 1260 | HC | 712.71979675 | 226.77723184 | 41 |
|  |  |  | PD | 831.65402778 | 344.82678170 | 24 |

**Table S6.** Response latencies (probe absent or present) for patients and controls on RSVP task. HC= Healthy Controls. PD = Parkinson’s disease.

As with our other analyses, we examined whether the results differed when we account for individual differences in performance. Data was missing or incomplete from 12 subjects on day 7 and these people were excluded from these control analyses. Adding overall day 7 accuracy as a normalised covariate into our analysis also revealed evidence for a significant interaction between drug and task (F(6,50) = 4.85, p = . 032, MSE = .013, η^2^_p_ = .09). Adding session in as a between-subject variable did not reveal any significant main effects of session or drug by session interactions (F<1). However, there was evidence for a significant three-way interaction between drug, AB interval and session (*F*(1,48) = 2.88, *MSE* = .011, *p* = .009, η^2^_p_ = .056). Post-hoc comparisons revealed a significant improvement in accuracy on caffeine (compared to decaffeinated performance) on the Decaffeinated/caffeine drug administration order for the longest (1260ms) Ab interval (*F*(1,48) = 23.35, *p* = .00004).

We examined whether individual differences in day 7 performance revealed additional drug effects. This was not the case. Including drug administration order as a between-subject variable in this model did not reveal a significant main effect of order (F(1,48) = 2.14, MSE = 666160, p = .14, η^2^_p_ = .042). However, there was evidence for a significant drug by order interaction (*F*(1, 48) = 4.447, MSE = 87866, p = .039, η^2^_p_ = .08). This was in the direction of caffeine slowing responses to the probe item if caffeine was administered on the first session, but the opposite tended to occur in the other drug order group, i.e., caffeine speeded responses if taken on the second session.
